# The analysis of segmental lordosis restored by oblique lumbar interbody fusion and related factors: building up preoperative predicting model

**DOI:** 10.1186/s12891-024-07293-5

**Published:** 2024-02-24

**Authors:** Jingye Wu, Tenghui Ge, Guanqing Li, Jintao Ao, Yuqing Sun

**Affiliations:** Department of Spine Surgery, Beijing Jishuitan Hospital, Capital Medical University, No. 31, Xinjiekou East Street, Xicheng District, Beijing, 100035 People’s Republic of China

**Keywords:** Segmental lordosis, OLIF, Predictive model, Deformity correction, Lumbar interbody fusion

## Abstract

**Background:**

Oblique lumbar interbody fusion (OLIF) procedures have the potential to increase the segmental lordosis by inserting lordotic cages, however, the amount of segmental lordosis (SL) changes can vary and is likely influenced by several factors, such as patient characteristics, radiographic parameters, and surgical techniques. The objective of this study was to analyze the impact of related factors on the amount of SL changes in OLIF procedures and to build up predictive model for SL changes.

**Methods:**

This is a retrospective study involving prospectively enrolled patients. A total of 119 patients with 174 segments undergoing OLIF procedure were included and analyzed. The lordotic cages used in all cases had 6-degree angle. Radiographic parameters including preoperative and postoperative segmental disc angle (SDA, preSDA and postSDA), SDA changes on flexion-extension views (ΔSDA-FE), CageLocation and CageInclination were measured by two observers. Interobserver reliability of measurements were ensured by analysis of interclass correlation coefficient (ICC > 0.75). Pearson correlation coefficient analysis and multivariate linear regression were employed to identify factors related to SDA changes and to build up predictive model for SDA changes.

**Results:**

The average change of segmental disc angle (ΔSDA, postSDA-preSDA) was 3.9° ± 4.8° (95% confidence interval [CI]: 3.1°-4.6°) with preSDA 5.3° ± 5.0°. ΔSDA was 10.8° ± 3.2° with negative preSDA (kyphotic), 5.0° ± 3.7° with preSDA ranging from 0° to 6°, and 1.0° ± 4.1° with preSDA> 6°. Correlation analysis revealed a significant negative correlation between ΔSDA and preSDA (*r* = − 0.713, *P* < 0.001), CageLocation (*r* = − 0.183, *P* = 0.016) and ΔSDA-FE (*r* = − 0.153, *P* = 0.044). In the multivariate linear regression, preSDA and CageLocation were included in the predictive model, resulting in minimal adjusted R^2^ change (0.017) by including CageLocation. Therefore, the recommended predictive model was ΔSDA = 7.9–0.8 × preSDA with acceptable fit. (adjusted R^2^ = 0.508, *n* = 174, *P* < 0.001).

**Conclusions:**

The restoration of segmental lordosis through OLIF largely depends on the preoperative segmental lordosis. The predictive model, which utilized preoperative segmental lordosis, facilitates preoperative planning for corrective surgery using the OLIF procedure.

## Background

Collapse of the intervertebral disc due to degenerative changes result in a decrease in segmental lordosis and disc height. For patients undergoing lumbar interbody fusion procedures, restoring optimal segmental lordosis at the index level(s) increases the lumbar lordosis and reduces the likelihood of adjacent segmental disease [1]. Various interbody fusion techniques using lateral approaches have the potential advantages of increasing segmental lordosis by inserting large-size interbody cages into intervertebral spaces [2–5]. Oblique lumbar interbody fusion (OLIF) developed a more oblique approach without splitting psoas and lumbar plexus injury, in comparison to the lateral lumbar interbody fusion (LLIF). OLIF had equivalent potential to increase the segmental lordosis and disc height on the sagittal plane [6, 7].

Mild to moderate loss of lumbar lordosis (PI-LL < 20°) can be effectively corrected by enlargement of segmental lordosis through OLIF procedures. The amount of correction of segmental lordosis at each level is better determined preoperatively, especially for patients with sagittal deformity, which is crucial for surgeons to choose the appropriate corrective strategies, including various osteotomy techniques. However, the precise magnitude of correction ability of OLIF has not been clarified yet and variable changes of segmental lordosis were observed in clinical practice. Only a few studies assessed the segmental correction by LLIF and demonstrated variable results, ranging from 2.8 to 5.0° changes of segmental lordosis [4, 5, 8].

This study was aimed to assess the amount of restoration of segmental lordosis by OLIF procedures and to identify related factors. Furthermore, the predictive models were aimed to be built up to estimate the correction of segmental lordosis preoperatively.

## Methods

### Patient population

A total of 139 consecutive patients with degenerative lumbar disorders and prospectively collected data, who underwent OLIF procedures with posterior fixations between July 2017 and August 2019 in the authors’ hospital, were retrospectively reviewed. Inclusion criteria included fused levels by OLIF within L2-L5, supplemented with posterior fixation, complete pre- and postoperative (within postoperative 1 week) lumbar spine radiographs and CT scans. Hybrid techniques of transforaminal interbody fusion (TLIF) and OLIF were also eligible for inclusion, but only the levels of OLIF were analyzed. Patients were excluded if they had moderate to severe lumbar degenerative scoliosis (Coronal Cobb angle of lumbar curve larger than 40°, which may interfere with the accuracy of measurements on lateral views), or if the fused level was isthmic spondylolisthesis or if they had vertebral body fractures or if posterior osteotomy was performed or if obvious intraoperative endplate injuries (greater than 2 mm endplate injury on lateral view) were observed. The operating surgeons were experienced with greater than 50 cases of OLIF. The study was approved by the ethical committee of the authors’ hospital.

Overall, 119 patients with 174 segments of OLIF were eligible for this study. Characteristic data, including age, gender, BMI, and diagnosis, together with the site and number of fused levels were listed in Table 1.

**Table 1:** Patients Characteristics

M/F	45/74
No. of Patients	119
Age (years, range)	62.1(33–86)
Total Levels	174
BMI	25.6 ± 3.0
Numbers of fused levels	
1	61
2	36
3	14
4	7
5	1
Fused Level	
L2/3	15
L3/4	47
L4/5	112
Diagnosis	
Degenerative spondylolisthesis	60
Degenerative spinal stenosis	44
Lumbar disc herniation	15

### Radiographic parameters and measurements

**Fig. 1:**
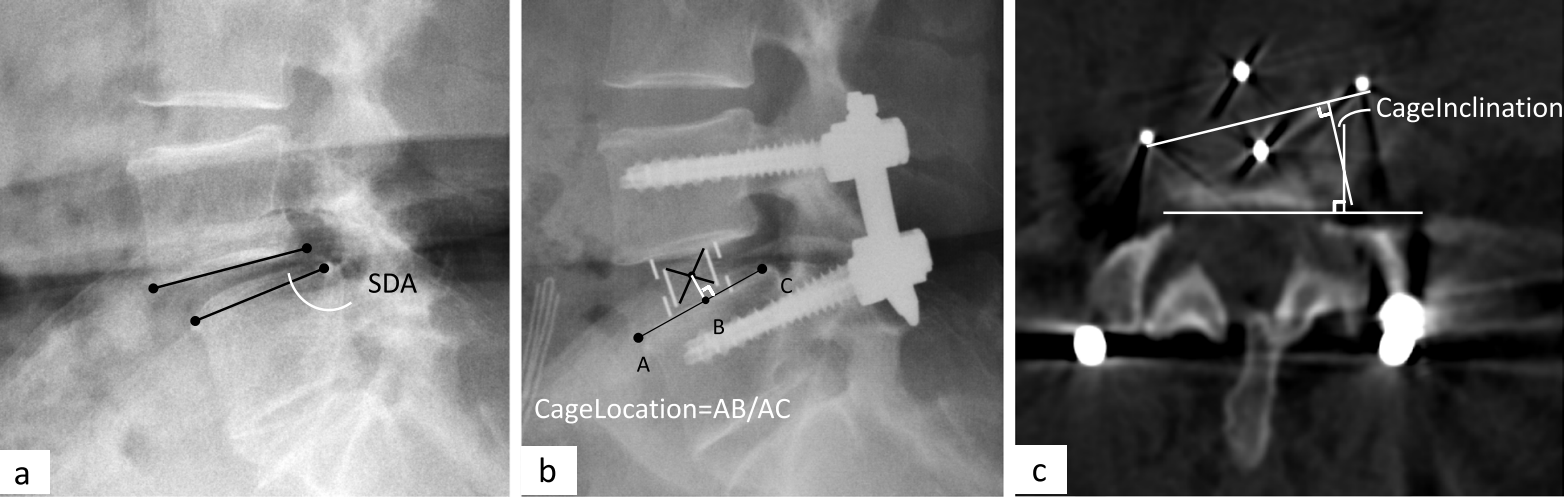
Definitions of radiographic parameters. **a** Segmental disc angle (SDA) on the standing lateral view of lumbar spine. **b** CageLocation, which is the ratio of AB to AC. B is the intersection point between perpendicular line from the midpoint of cage and the upper endplate. A and C are the anterior and posterior margin of upper endplate. **c** CageInclination, which is the angulation between axis of cage and posterior border of vertebral body

Change of segmental disc angle (ΔSDA) is defined as the postoperative segmental disc angle (postSDA) minus the preoperative segmental disc angle (preSDA), representing the angulation between cranial and caudal endplates at disc level of interest. A negative value indicates kyphosis at the level. SDAs were measured on the standing lateral radiograph which was obtained within postoperative 7 days. The measurement illustration was presented in Fig. 1a.

- ♦ ΔSDA-FE: SDA changes assessed on flexion-extension radiographs, calculated as SDA on extension minus SDA on flexion.
- ♦ CageLocation, referring to the ratio of distance from cage midpoint to anterior margin of upper endplate and length of upper endplate on lateral radiograph (Fig. 1b).
- ♦ CageInclination: the angulation between cage axis and posterior border of vertebral body on axial view of CT scans (Fig. 1c).

All radiographic measurements were conducted on Carestream PACS (Version 11.0) by two independent observers (T.H.G. and G.Q.L.) who underwent initial measurement training by senior surgeons. They demonstrated good inter-observer agreements (interclass correlation coefficient, ICC > 0.75) in the first 30 measurements for each parameter mentioned above. Once all the measurements were completed, the values of two observations were averaged if ICC > 0.75.

### OLIF procedure

OLIF procedures were performed following the manual of Medtronic OLIF25 [9]. No anterior longitudinal ligament release was performed. Appropriate size of cage with 6 degrees of lordosis (Clydesdale Spinal System, Medtronic) was selected and inserted into the proper position which was confirmed under fluoroscopy. The bone grafts inside the cage were allograft mixed with demineralized bone matrix (AlloMatrix, Wright Medical). Anterior placement of cage was attempted for maximal segmental lordosis.

Posterior pedicle screw fixation was performed for all cases after changing position into prone position. Percutaneous pedicle screw fixation was performed if adequate indirect neural decompression could be achieved, otherwise open direct neural decompression, either by laminectomy or laminotomy with pedicle screw fixation was performed. No compressive force across pedicle screw heads to increase segmental lordosis was applied for all patients.

### Statistic method

Correlations between outcome measures (ΔSDA) and predictive variables were analyzed by using one-way ANOVA or independent samples *t* test (for categorical predictive variables through between-group comparisons) or Pearson correlation coefficient (for continuous predictive variables). The predictive variables included level, numbers of fused level, decompression method (indirect versus direct) and radiographic parameters mentioned above. Multivariate linear regression with stepwise regression of independent variables was used to build up the predictive model for ΔSDA. Interobserver agreements for measurement of each radiographic parameter were assessed by the analysis of ICC [10].

SPSS (version 23.0) was used for the statistical analysis. The statistically significant level of difference was assumed at *P* < 0.05 based on two-side hypothesis test.

## Results

Good interobserver agreements of radiographic measurement were observed as the ICC was greater than 0.75 for each radiographic parameter.

For changes of segmental lordosis, the total average value of ΔSDA was 3.9° ± 4.8° (95% confidence interval [CI]: 3.1°-4.6°) with preSDA 5.3° ± 5.0°. There were no significant differences of ΔSDA across different levels(*P* = 0.285) and numbers of fused levels (*P* = 0.126) based on the one-way ANOVA, indicating no correlation between levels or numbers of fused levels and ΔSDA. See Table 2. The values of ΔSDA in direct and indirect decompression groups (96 versus 78 segments, 3.6° versus 4.2°) revealed no significant differences according to the independent sample *t* test. (*t* value = − 0.785, *P* = 0.434).

**Table 2:** ΔSDA at different levels and with different numbers of fused levels

	Level			Numbers of fused levels			Total
	L2/3	L3/4	L4/5	1	2	≥3	
Total	15	47	112	61	59	54	174
preSDA (°)	4.1 ± 4.5	5.4 ± 4.3	5.4 ± 4.6	5.2 ± 4.5	5.9 ± 4.9	4.6 ± 4.0	5.3 ± 5.0
postSDA (°)	8.7 ± 2.2	8.3 ± 3.2	9.6 ± 3.8	9.5 ± 3.7	8.8 ± 3.6	9.1 ± 3.3	9.1 ± 3.6
ΔSDA (°)	4.6 ± 4.3	2.9 ± 3.9	4.2 ± 5.2	4.3 ± 5.2	2.8 ± 5.2	4.5 ± 3.7	3.9 ± 4.8
95% CI (°)	2.3–7.0	1.8–4.1	3.2–5.1	3.0–5.6	1.5–4.2	3.5–5.5	3.1–4.6
Minimum value (°)	−3.2	−7.2	−8.1	−4.8	− 8.1	−5.4	−8.1
Maximum value (°)	11.3	10.0	17.2	16.3	17.2	13.3	17.2
*F* value*	1.266			2.098			NA
*P* value†	0.285			0.126			NA

* *F* value in one-way ANOVA

† Comparisons of difference levels and numbers of fused levels by ANOVA analysis

*SDA* Segmental disc angle, *preSDA, postSDA* Preoperative and postoperative SDA, *ΔSDA* Change of SDA

### Correlation analysis of related factors with ΔSDA

Correlation analysis revealed the ΔSDA had significant negative correlation to preSDA (*r* = − 0.713, *P* < 0.001), CageLocation (*r* = − 0.183, *P* = 0.016) and ΔSDA-FE (*r* = − 0.153, *P* = 0.044). as shown in Table 3.

**Table 3:** Pearson correlation analysis between predicting variables and ΔSDA

		Average Value	*P* Value	Correlation Coefficient(r)
ΔSDA	preSDA (°)	5.3 ± 5.0	0.000	−0.713
	ΔSDA F-E (°)^a^	3.1 ± 1.9	0.044	−0.153
	CageLocation	0.45 ± 0.08	0.016	−0.183
	CageInclination (°)	7.4 ± 4.3	0.274	NA

^a^Means SDA changes on flexion-extension views of lumbar radiographs. *SDA* Segmental disc angle, *preSDA* Preoperative SDA

**Fig. 2:**
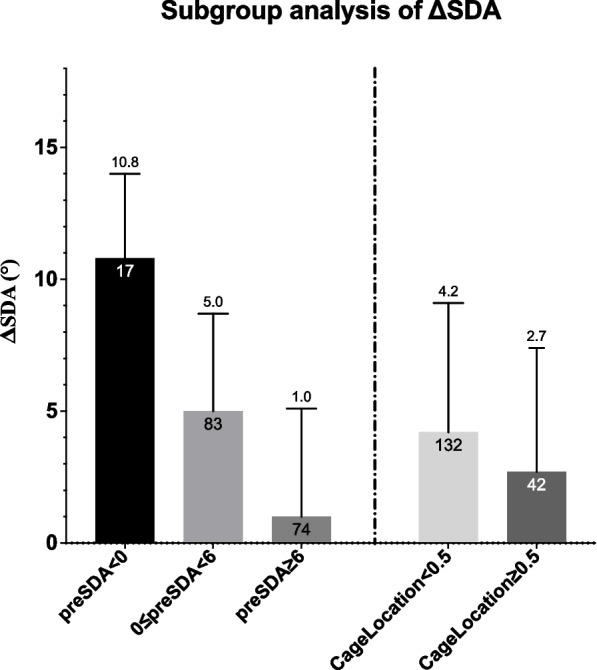
Subgroup analysis of different preSDA and CageLocation affecting ΔSDA. preSDA: preoperative segmental disc angle; ΔSDA: SDA change

**Fig. 3:**
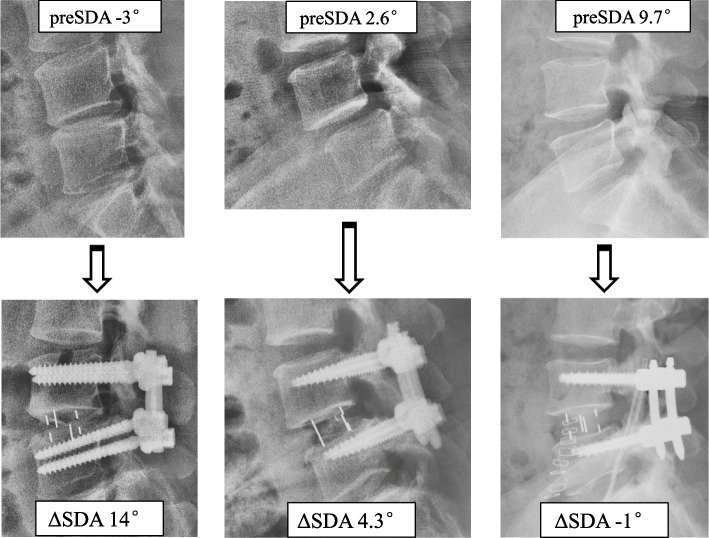
Case examples of SDA changes at L4/5 level. SDA: segmental disc angle

**Fig. 4:**
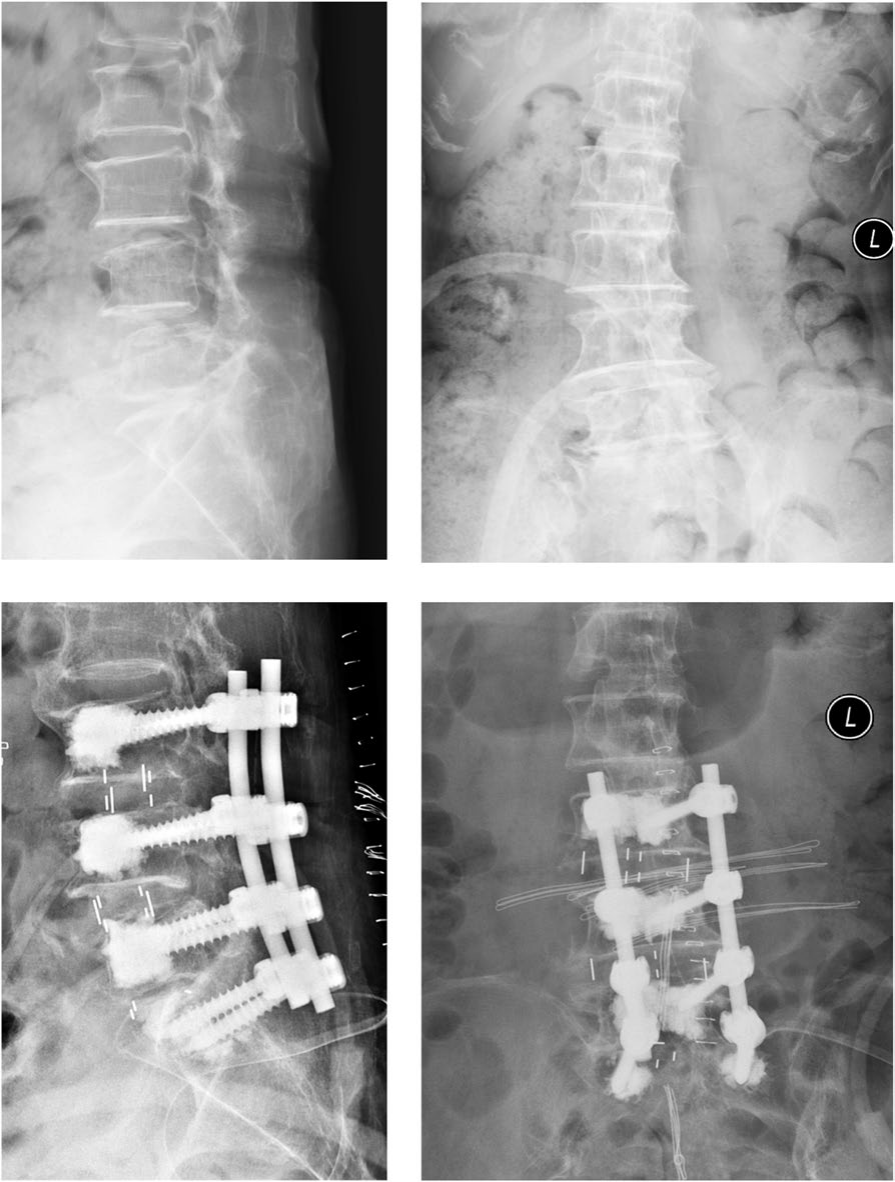
This is a 77-year-old female patient who complained of radiating pain over bilateral buttocks anterior thigh and lower limbs. Image studies showed L4 spondylolisthesis, L3–4 and L5-S1 spinal stenosis. Neural decompression and L3-S1 interbody fusion were performed with L3–5 OLIF and L5-S1 TLIF. ΔSDA:1.5° at L3–4 level, 7.8° at L4–5 level

Subgroups of different preSDA and CageLocation were analyzed (see Fig. 2). Among the three preSDA subgroups, ΔSDA was 10.8° ± 3.2° with negative preSDA (indicating kyphotic), 5.0° ± 3.7° with preSDA ranging from 0° to 6°, and 1.0° ± 4.1° with preSDA> 6°. Among the two CageLocation subgroups, ΔSDA was 4.2° ± 4.9° with CageLocation< 0.5 (indicating anteriorly placed), and 2.7° ± 4.7° with CageLocation≥0.5. Case examples of different preSDA were shown in Fig. 3 and a case example was shown in Fig. 4.

### Building the predictive model for ΔSDA

Multivariate linear regression was employed to predict ΔSDA. The independent variables, including preSDA, ΔSDA-FE, CageLocation and CageInclination were entered into the regression model using the stepwise multiple regression method. ΔSDA-FE and CageInclination were removed through this method of regression.

The analysis indicated that the predictive model ONE utilizing two variables, preSDA and CageLocation, was a robust model with an adjusted R^2^ of 0.522. The predictive model was presented as ΔSDA =11.9–0.8 × preSDA-9 × CageLocation (*P* = 0.008, *n* = 174). Predictive model TWO was presented as ΔSDA =7.9–0.8 × preSDA (adjusted R^2^ 0.505, *P* < 0.001, *n* = 174) by including only preSDA. The adjusted R^2^ change was only 0.017 between model ONE and TWO, suggesting a minimal effect of CageLocation on ΔSDA. As indicated by the predictive models, a smaller preoperative segmental disc angle and a more anteriorly placed cage could lead to larger postoperative segmental disc angles, and vice versa. Detailed results were presented in Table 4.

**Table 4:** Predicting models for ΔSDA by multiple regression analysis

Variables	Unstandardized Coefficients-B	Standard Error	Standardized Coefficients-Beta	*t* Value	*P* Value	Adjusted R^2^
Model ONE						
Intercept	11.881	1.529		7.769	0.008	0.522
preSDA	−0.76	0.057	−0.704	−13.377		
CageLocation	−8.956	3.343	−0.141	−2.679		
Model TWO						
Intercept	7.921	0.399		19.838	< 0.001	0.505
preSDA	−0.769	0.058	− 0.713	−13.325		

*SDA* Segmental disc angle, *preSDA* Preoperative SDA, *ΔSDA* Change of SDA

## Discussion

Restoring segmental lordosis during lumbar interbody fusion is necessary, as optimal lumbar lordosis was crucial for sagittal balance and restoring adequate segmental lordosis reduces the likelihood of adjacent segment disease [1]. Moreover, for patients requiring corrective surgery on the sagittal plane, estimating the amount of correction through specific technique, such as osteotomy or cage insertion, is essential for surgeons to choose optimal corrective strategies preoperatively [11]. If angular correction by cage insertion is sufficient, posterior osteotomy can be avoided, as open osteotomy procedures are associated with massive blood loss and increased morbidities especially for elderly patients [12].

During OLIF procedure, the placement of a large lordotic cage into intervertebral space can significantly reverse the collapse of the disc, thus regaining the disc height and segmental lordosis through a minimally invasive approach. These are the main advantage for lateral approaches of the lumbar interbody fusion [5]. However, the postoperative disc angle was not equal to the lordotic cage angle (in this study, postSDA 9.1° ± 3.6°), due to the lack of full contact of cage on the concave endplates with most contact occurring at the anterior margin of cage [2]. Therefore, the amount of correction achieved by lateral approaches of interbody fusion varies and has its limits.

The predictive model (ΔSDA =7.9–0.8 × preSDA) was developed in this study, which highlights the preoperative factor preSDA significantly determines the postoperative segmental lordosis. This means that, during preoperative planning, surgeons can use this model to estimate the amount of angular correction at the disc level(s) through OLIF. This model provides practical information during decision-making process.

### Segmental lordosis corrected by OLIF and possible related factors

The mean correction of SDA in this study was 3.9° (95% CI: 3.1°-4.6°) at each level. Previous studies revealed similar correction magnitude of SDA by OLIF, ranging from 4.5° to 5.1° on average [13–15]. This study identified three potential factors that affect angular correction by OLIF: preoperative SDA, Cage location and SDA changes on flexion-extension views.

As an uncontrolled factor by surgeons, preoperative SDA was the strong predictor for the change of SDA (*r* = − 0.713). This strong correlation was similarly described by other studies of LLIF [2, 4]. Uribe et al also revealed significant relationship between preoperative SDA and changes of SDA in a literature review of variable techniques of lumbar interbody fusion, including TLIF [16]. The larger the preoperative SDA, the less the amount of postoperative SDA would increase. Nevertheless, this effect has its limits. The reason why SDA was difficult to further increase could be the tightness of surrounding ligamentous structures, especially the anterior longitudinal ligament, restricting the lift-up of the disc space. This limitation effect was diminished by the release of anterior longitudinal ligament even if a large preoperative SDA exists [17, 18].

Cage location, a factor controlled by surgeons, was confirmed to be another predictive factor in this study. Otsuki et al analyzed the factors affecting the SDA by LLIF at a total of 102 levels and found that cage location affected the changes of SDA [2]. An anteriorly placed cage had a larger amount of correction of SDA than a posteriorly placed cage. Besides, anterior placement maintains the effect of indirect decompression for intervertebral foramen or central canal [19]. This means it’s better to place cage anteriorly to gain more lordosis to maximize the correction capacity of cage insertion. However, the effect of cage location on ΔSDA was minimal as the between-group difference was merely 1.5° (anterior versus posterior cage location) and changes of adjusted R^2^ was only 0.017 by including CageLocation as the predictive variable in the regression model.

Given the minimal effect of cage location on ΔSDA, the predictive model only including preoperative SDA was built up. This model can assist surgeons in predicting the amount of SDA change preoperatively for the segment of interest using a single factor, the preoperative SDA.

Another affecting factor was SDA changes on flexion-extension views, which indicated the segmental flexibility. Theoretically, more rigid segment would have less amount of correction, which can be indicated by SDA changes on flexion-extension views. The correlation analysis in this study showed significant but weak correlations (*r* = − 0.153) and further multivariate linear regression analysis removed it to build up predictive model. The reason why this correction was weak could be radiating pain or low back pain result in less segmental mobility on flexion-extension views, which may underestimate the actual segmental flexibility. Yen et al [5] found that intradiscal vacuum phenomenon, an indicator of segmental instability and high mobility, was a predictive factor of SDA changes in LLIF, suggesting the segmental flexibility could be indicated by intradiscal vacuum phenomenon.

Other possible factors, including level, total number of fused levels, CageInclination, decompression procedure, were also analyzed and demonstrated no correlation with SDA changes. This result suggested there were no difference of SDA changes across the levels, no influence by adjacent fused levels. Meanwhile, the obliquity of cage placement and decompression procedure had no effect on SDA changes.

### Limitations

In this study, the lordotic angle of cages were all 6 degrees. Cages with larger lordotic angle (such as 10 or 12 degrees) may have greater amount of correction of SDA for flexible segments or large preoperative SDA (> 6 degrees). However, as mentioned above, the tightness of anterior ligaments may restrict the effect of angular correction, reaching its limit. With adequate lift-up of disc space by the anterior margin of cage, greater lordotic design cannot further increase the SDA without ALL release, as posterior margin of cage may have no contact with endplates, which was shown in a study of LLIF [2]. Further increasing cage size and force impaction may cause endplate injury or vertebral fracture. Therefore, larger lordotic cages may not result in larger angular correction than 6-degree cages, especially for rigid segments.

Another limitation of this study was the lack of long-term observation of SDA changes. During follow-up, cage migration or subsidence may jeopardize the sagittal angular correction by OLIF, however, this complication occurred less with posterior fixation than with the stand-alone technique [20].

The development of a predictive model (ΔSDA = 7.9–0.8 × preSDA) is a significant contribution of this study. Due to the nature of a single-center study, the applicability may be influenced by different patient populations, variations in surgical techniques, or preferences of surgeons. It is advisable to consider revisions to this model when applied in different centers.

## Conclusions

The restoration of segmental lordosis through OLIF largely depends on preoperative segmental lordosis. The recommended predictive model was ΔSDA = 7.9–0.8 × preSDA. The predictive model, which utilize the preoperative segmental lordosis facilitates preoperative planning for corrective surgery using the OLIF procedure.

## Data Availability

No datasets were generated or analysed during the current study.

## Abbreviations

OLIF: Oblique lumbar interbody fusion
SL: Segmental lordosis
SDA: Segmental disc angle
ΔSDA: Changes of segmental disc angle
LLIF: Lateral lumbar interbody fusion
ALL: Anterior longitudinal ligament
ICC: Interclass correlation coefficient
